# LCDAE: Data Augmented Ensemble Framework for Lung Cancer Classification

**DOI:** 10.1177/15330338221124372

**Published:** 2022-09-23

**Authors:** Zeyu Ren, Yudong Zhang, Shuihua Wang

**Affiliations:** School of Computing and Mathematical Sciences, 4488 University of Leicester, Leicester LE1 7RH, UK

**Keywords:** machine learning, medical image analysis, generative adversarial networks, ensemble

## Abstract

**Objective:** The only possible solution to increase the patients’ fatality rate is lung cancer early-stage detection. Recently, deep learning techniques became the most promising methods in medical image analysis compared with other numerous computer-aided diagnostic techniques. However, deep learning models always get lower performance when the model is overfitting. **Methods:** We present a Lung Cancer Data Augmented Ensemble (LCDAE) framework to solve the overfitting and lower performance problems in the lung cancer classification tasks. The LCDAE has 3 parts: The Lung Cancer Deep Convolutional GAN, which can synthesize images of lung cancer; A Data Augmented Ensemble model (DA-ENM), which ensembled 6 fine-tuned transfer learning models for training, testing, and validating on a lung cancer dataset; The third part is a Hybrid Data Augmentation (HDA) which combines all the data augmentation techniques in the LCDAE. **Results:** By comparing with existing state-of-the-art methods, the LCDAE obtains the best accuracy of 99.99%, the precision of 99.99%, and the F1-score of 99.99%. **Conclusion:** Our proposed LCDAE can overcome the overfitting issue for the lung cancer classification tasks by applying different data augmentation techniques, our method also has the best performance compared to state-of-the-art approaches.

## Introduction

Cancers are a major cause of mortality worldwide, there are accounted to be around 10 million deaths in 2020.^1^ The cancers can exist in different organs. For example, the brain, lungs, liver, stomach, colon, skin, and prostate.^2–4^ They also have different xenogeneses: daily habits such as smoking and alcohol intake; the ultraviolet and radiation are regarded as physical carcinogens; moreover, the chemical carcinogens such as genetic and biological carcinogens.^1^ If cancer has not been treated in the early stage, most cancer cells will eventually become uncontrollable and spread throughout the different organs as time goes on.^5^ Among the various types of cancers, lung, colon, and rectum cancers caused the most deaths for males and females. In 2020, there are around 2.21 million new cases reported as lung cancers and more than 1.8 million deaths due to lung cancers worldwide.^1^ Fortunately, if cancer can be diagnosed at early stages, the survival rate of patients will increase, and the patients also have enough time to do treatment. However, there are only 20% of patients are diagnosed in the early stage of lung cancer,^6–8^ and using traditional diagnosis methods to detect lung cancer in the early stage is difficult.

Computer-aided diagnosis systems aim to support doctors in analyzing medical images and making decisions quickly.^9^ In the last few decades, deep learning has been a common method in medical image analysis for pattern recognition, image classification, and image segmentation.^10–13^ However, overfitting problems are common in deep learning models. The main reasons behind the overfitting are various: presence of noise, limited size of training data, and complexity of models.^14^ Especially in medical image analysis, lacking medical training datasets is a common problem, and the data features are not easy to present like in other domains. Moreover, most models cannot perform excellently on medical image datasets. It is better to concatenate different models or do fine-tuning to get higher performance for the medical image analysis tasks. Therefore, to resolve these challenges, we proposed a Lung Cancer GAN based Ensemble (LCDAE) framework. There are 3 parts of the LCDAE. Firstly, a Lung Cancer Deep Convolutional GAN (LDCGAN) can make 3 types of artificial lung cancer images. Another part is a data augmented ensemble model called DA-ENM. The DA-ENM uses 6 pretrained models: DenseNet121, GoogleNet, ResNet101, VGG19-BN, VGG16-BN, and VGG16. The last part is a Hybrid Data Augmentation (HDA) which combines all the data augmentation techniques in the LCDAE, it includes LDCGAN and other data augmentation techniques in the DA-ENM. In order to solve the overfitting problem. Firstly we use generated synthetic lung cancer images from LDCGAN as an additional training dataset to train the DA-ENM. Secondly, we use multiple data augmentation techniques to increase the generalizability of our framework. Thirdly, before we create the ensemble model, we use fine-tuning techniques for all the pretrained models to learn the higher-order feature representations and improve the performance.

We analyze the results of LCDAE with existing state-of-the-art approaches. Our method reaches the highest accuracy of 99.99%, the precision of 99.99%, and the F1-score of 99.99%, except the sensitivity of 99.99%, which is a bit lower than the highest one, 100%. The contributions are summarized as follows:

- A data augmented ensemble framework LCDAE is introduced to classify different classes of lung cancer datasets and also overcome the overfitting issues. The ensemble model can concatenate different submodels, which can help the model to explore the hidden data features of the dataset.
- A LDCGAN can produce artificial lung cancer images, and the model solves the problem of the small number of medical images by generating synthetic pictures. The synthesized medical images also can help the model to get higher performance.
- A Hybrid Data Augmentation (HDA) can increase the generalizability and overcome the overfitting problem of the model. It also can prevent data scarcity and reduce the cost of collecting labeled data.
- Our method gets the best performance compared with existing up-to-date methods: 99.99% (accuracy), 99.99% (precision), 99.99% (F1-score), and 99.99% (sensitivity).

## Related Work

### Lung Cancer Classification

In 2022, Patra et al^15^ proposed a Deep Maxout Network with Dolphin-based Henry Gas Solubility Optimization. Firstly, they used a Gaussian filter. Secondly, the RoI extraction is also used for the image preprocessing, then using the U-Net model to generate segments to do classification, the final results have accuracy of 93.08% and sensitivity of 94.81%.

A novel DL-based supervised learning method was proposed by Masud et al^5^ to classify 5 types of lung cancer tissues. This approach applied 2 feature extraction methods: 2D Fourier Features and 2D Wavelet Features. Then they concatenated 2 domain transformations to build the final resultant features. In the end, their work reached 96.33% (accuracy) and 96.38% (F-measure score).

Shakeel et al^16^ have developed a method, which achieved a 2.12% of minimum error rate and 99.48% of prediction rate. Firstly, they normalize the original data and then examine the redundant features before fed into the AdaBoost optimized ensemble learning generalized neural network.

Lakshmanaprabu et al^17^ introduced the Optimal Deep Neural Network with additional hidden layers to classify the lung computed tomography (CT) images. They also proposed a Linear Discriminate Analysis to decrease the features’ dimensions. The developed technique got the results as follows: 94.56% (accuracy), 96.2% (sensitivity), and 94.2% (specificity).

Khan et al^18^ proposed a contrast-based feature fusion and selection method for the lung cancer CT image classification tasks. In this proposed method, they used gamma correction max intensity weights (GCmIW) to enhance the contrast, and they also used a serially canonical correlation-based method to fuse the multiple feature maps. Finally, this approach reached an accuracy of 99.4% on the Lungs Data Science Bowl 2017 dataset. In 2019, Khan et al^19^ also introduced a Lungs nodule detection framework with a support vector machine, the proposed framework used several data augmentation techniques such as contrast enhancement and feature extraction, and this work got a sensitivity of 97.45% on the Lungs Image Consortium Database dataset.

Moreover, there are related works using ensemble for the classification tasks, such as Onan et al.^20–23^ And other different models for the classification tasksin Onan^24^ and Onan and Korukoğlu,^25^ Further related works also related to the background of this research are in the literature.^26–32^

### Avoid Overfitting for Deep Learning

Deep learning models have shown powerful performance on computer vision applications and tasks. However, the big dataset is always the key part of models to present overfitting problems. Overfitting refers to a function of the model which is exactly fitted against its limited aligned data, and the function cannot perform well on the new dataset.^33^ There are various ways to avoid the overfitting problem in deep learning. Here we summarize them as 3 different parts: data augmentation, model architectures, and functional solutions.

#### Data Augmentation

Data augmentation includes a series of approaches that focus on enhancing the size and quality of the original dataset in order to provide sufficient high-quality training images to train the neural network. Here, we summarize all the data augmentation techniques into 2 categories. Initially, we will explain the basic image manipulations. Then we will discuss the approaches based on deep learning.

Basic image manipulations are well used for most deep learning applications. Firstly, the geometric transformations which include flipping, color space transformations, cropping, rotation, translation, and noise injection.^33^ These geometric transformation techniques are very efficient to deploy in the deep learning models. The next one is the kernel filter. It is the most classical method to sharpen and blur images. Kang et al^34^ used a unique kernel filter technique called PatchShuffle Regularization. It reached an accuracy of 94.34% on the CIFAR-10 dataset. The third one is mixing images. According to the experiments conducted by Inoue^35^, they calculate the mean of pixel values for each channel of the image, which can be regarded as an efficient augmentation method. The random erasing developed by Zhong et al^36^ is another efficient technique inspired by dropout regularization. It will randomly erase certain features of images in the entire dataset. The technique proves its feasibility when dealing with the occlusion problem of image recognition tasks. The last one is combining different basic image manipulations based on the demands of the deep learning applications.

Data augmentation based on deep learning approaches is also a promising way to be implemented in image analysis applications. These approaches can be divided into 4 types: neural style transfer; adversarial training; augmentation in feature space, and GAN-based data augmentation.^33^ The primary image manipulations are focused on the input space. Instead, the feature space augmentation will focus on the lower-dimensional feature representations. Terrance and Graham^37^ discussed the augmentation technique in the feature space. Adversarial training is a machine learning technique that uses obtainable models to create malicious attacks.^38^ Adversarial attacking is one of the most common techniques used in adversarial training frameworks. The adversarial attacking has multiple rival networks to learn the data augmentations for the misclassified images.^33^ The third one is GAN-based data augmentation. It has an impressive performance to produce additional datasets and get better performance for the image classification and segmentation tasks. Finally, the neural style transfer^39^ is also a potential data augmentation technique to transfer the style of the source image to the target image, it gets excellent success for the artwork domain.

#### Model Architectures

Apart from the data augmentation techniques, some strategies also focus on the model architecture itself to avoid overfitting and improve model’s generalization ability. There already exists various model architectures which proved to be the reliable model architectures such as LeNet-5,^40^ AlexNet,^41^ GoogLeNet,^42^ ResNet,^43^ VGG16,^44^ and DenseNet.^45^

#### Functional Solutions

There already exist many successful functional solutions in many deep learning applications, such as layer normalization, batch normalization, and dropout regularization. Beyond basic functional solutions, the transfer learning aims to use pretrained models to get better performance across similar domains.^46^ Meta learning can evaluate the differences between different machine learning models deployed on new tasks or new domains with less training examples, it is also known as “learning to learn.”^47^ Ensemble is also an efficient way to get better generalization ability by combining different predictions from the multiple models to decide the final prediction.^48^

## Methods

### Dataset

**Figure 1.**
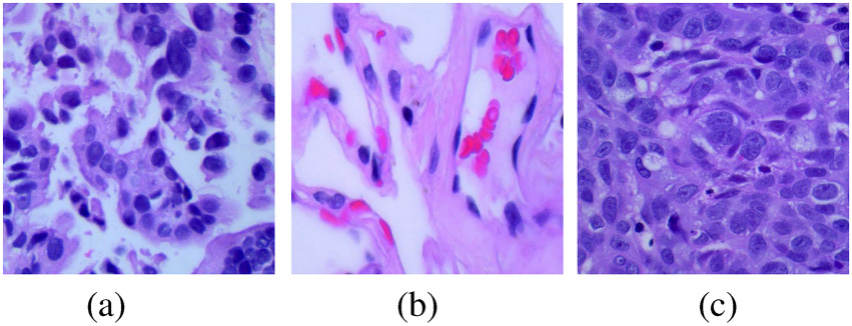
The example of (a) lung adenocarcinoma, (b) lung benign, and (c) lung squamous cell carcinoma from the raw dataset.

The dataset includes 15 000 histopathological lung cancer images^68^ with 3 classes: lung adenocarcinoma, lung benign, and lung squamous cell carcinoma. For each class, there are 5000 images. The original size of the images is 768×768 in jpeg format. The example of lung adenocarcinoma is shown in Figure 1(a), lung benign in Figure 1(b) and lung squamous cell carcinoma in Figure 1(c). Overall, our original dataset includes 3 classes and 5000 images for each class, in total 15 000 images.

### Proposed Data Augmented Ensemble LCDAE Framework

**Figure 2.**
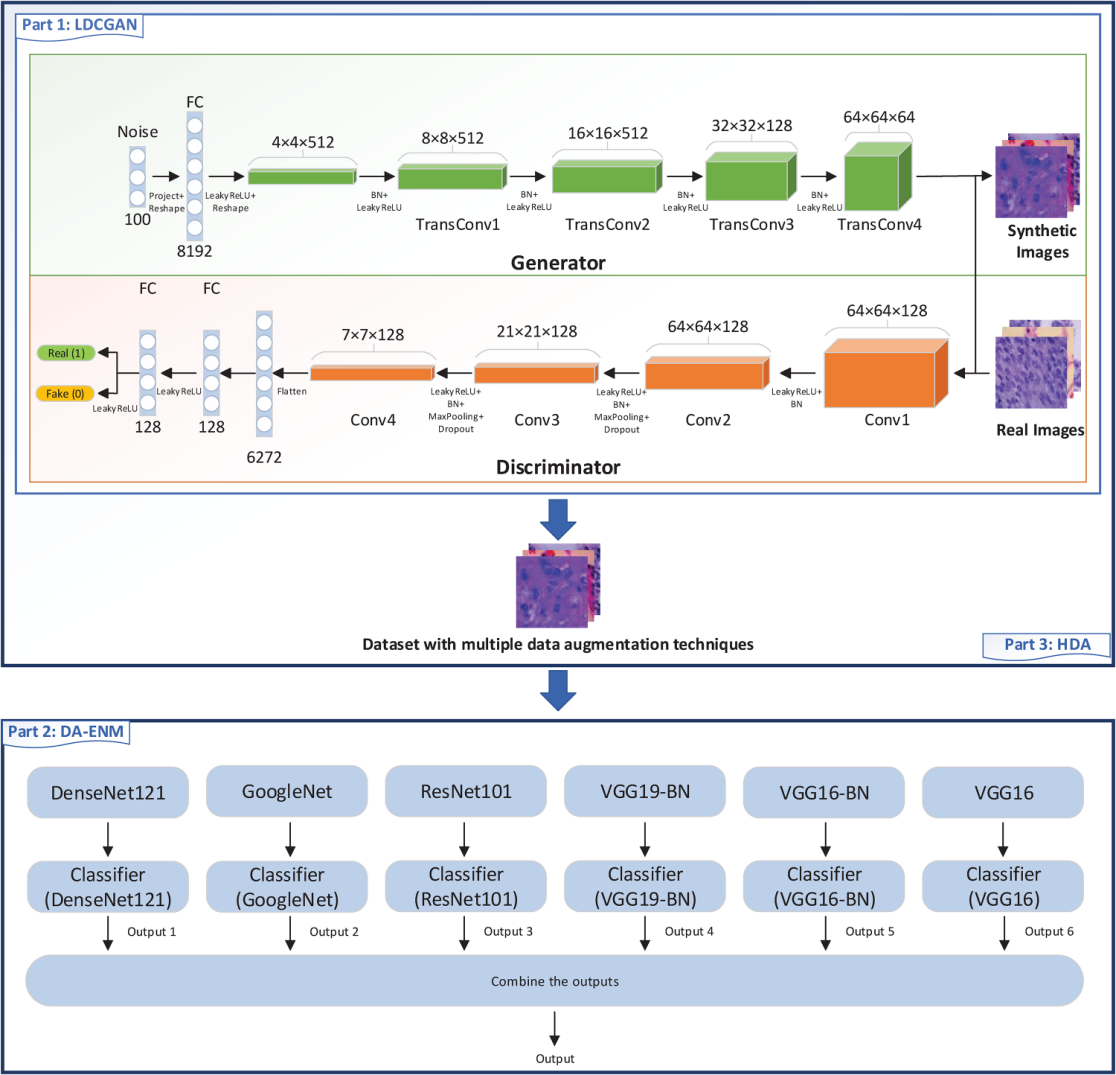
The architecture of LCDAE.

Our proposed LCDAE framework consists of 3 parts: the generative model LDCGAN; data augmented ensemble model DA-ENM, and hybrid data augmentation (HDA). We will discuss the generative model LDCGAN in section “Generative Model LDCGAN”, the data augmented ensemble model DA-ENM in section “Data Augmented Ensemble Model DA-ENM” and hybrid data augmentation (HDA) in section “Hybrid Data Augmentation”. The entire architecture of LCDAE is shown in Figure 2.

#### Generative Model LDCGAN

The LDCGAN is inspired by the DCGAN,^50^ and we tailored it to classify different types of lung cancer images. The LDCGAN has 2 components. The first one is a generator *G*, and the second one is a discriminator *D*. It belongs to unsupervised learning, which consists of traditional convolutional architecture with additional constraints. The generator *G* is trying to obtain the features from the original dataset and generate a synthetic dataset. The discriminator *D* is trained to predict the outputs generated from the *G*, whether they are real or artificial images. Both *G* and *D* compete with each other, the *G* trying to generate fake images as similar to the real images as possible, then trying to make *D*’s prediction to be wrong. The role of *D* is trained to improve the accuracy of distinguishing authentic and artificial images. The idea of GAN comes from the minimax algorithm in game theory. Finally, the *G* is well trained to produce synthetic lung cancer images when the *D* cannot find the differences between artificial images and authentic images. The mathematical representation will be illustrated in the next paragraph.

We use $p_{g}$ to present the probability distribution of the generator. The x is training data and the input noise is $p_{z}(z)$ . The generator’s parameters are indicated by $\theta_{g}$ , and $G(z,\theta_{g})$ is the data space with parameters $\theta_{g}$ , which comes from the input noise $p_{z}(z)$ .

Equation (1) shows the principle behind the training process of GAN. Firstly, the generator *G* tries to decrease the loss L(D,G) , and train the discriminator *D* to maximize it. After the training process, we can get a well-trained generator to produce synthesized images that the discriminator cannot predict whether images are real or fake. The entire process can be regarded as a minimax game between *D* and *G*. In this game, *G* tries to minimize the chance that the *D* will output results as synthetic images, the probability represented by the log(1−D(G(z))) , and *D* will try to improve the probability that it can make the right predictions logD(x) . Overall, equation (1) describes the loss function of the GAN.

(1) $$\begin{array}{cc} \min_{G}\max_{D}V(D,G) & =E_{x∼p_{data}(x)}\left[\log\;D(x)\right]\\ & +E_{z∼p_{z}(z)}\left[\log\;(1-D(G(z)))\right] \end{array}$$

Apart from the default setting for the original DCGAN, we use some additional settings to avoid checkerboard patterns and overfitting problems:

1. Add more filters for each layer, especially for the front layers of the generator. The additional filters can help the generator capture more original image features and avoid producing blurry images.
2. Add additional dropout layers to the structure of the discriminator. These dropout layers can avoid the overfitting problem.
3. According to the experiments by Shi et al^51^, we make that the size of the kernel is divisible by the stride. Moreover, set the maximal kernel size. The biggest kernel size can capture the features in the front layers, and it can solve checkerboard patterns.
4. Use a bilinear interpolation algorithm during the process of resizing to avoid checkerboard patterns of produced images. The algorithm of bilinear interpolation shows in the equation (2). Here, we use a 2D bilinear interpolation algorithm on 2 axes (*x* and *y* axes). We define 4 locations: $\left(i_{1},j_{1}\right)$ of $P_{11}$ , $\left(i_{1},j_{2}\right)$ of $P_{12}$ , $\left(i_{2},j_{1}\right)$ of $P_{21}$ and $\left(i_{2},j_{2}\right)$ of $P_{22}$ . In the rectangle defined by these locations, we can calculate any location (i,j) which is inside the rectangle.
5. Additional batch normalization^52^ layers are added to the generator and discriminator. These batch normalization layers can standardizes inputs of the current layer, and help models avoid overfitting problems. The algorithm of batch normalization is shown in equation (3). In this algorithm, τ is a batch of training data, and it has n training examples. Firstly, we calculate the mean of the current batch. Secondly, the variance is calculated. Then the algorithm normalizes $x_{j}$ . Finally, it scales and shifts the final result.

(2) $$\begin{array}{cc} \;f(i,j)\approx & \frac{\;f(P_{11})}{\left(i_{2}-i_{1})(j_{2}-j_{1}\right)}(i_{2}-i)(j_{2}-j)\\ & \;+\frac{\;f(P_{21})}{\left(i_{2}-i_{1})(j_{2}-j_{1}\right)}(i-i_{1})(j_{2}-j)\\ & \;+\frac{\;f(P_{12})}{\left(i_{2}-i_{1})(j_{2}-j_{1}\right)}(i_{2}-i)(j-j_{1})\\ & \;+\frac{\;f(P_{22})}{\left(i_{2}-i_{1})(j_{2}-j_{1}\right)}(i-i_{1})(j-j_{1}) \end{array}$$

(3) $$\begin{array}{ccc} & \eta_{\tau }\leftarrow \frac{1}{n}\sum_{\;j=1}^{n}x_{\;j} & \;\;\text{mean\textbackslash{};of\textbackslash{};the\textbackslash{};batch}\\ & \nu_{\tau }^{2}\leftarrow \frac{1}{n}\sum_{\;j=1}^{n}(x_{\;j}-\eta_{\tau }{)}^{2} & \;\;\text{variance\textbackslash{};of\textbackslash{};the\textbackslash{};batch}\\ & \hat{x_{j}}\leftarrow \frac{x_{j}-\eta_{\tau }}{\sqrt{\nu_{\tau }^{2}+\epsilon }} & \;\;\text{normalization},\\ & y_{j}\leftarrow \gamma \hat{x_{j}}+\phi \equiv {\mathrm{BN}}_{\gamma ,\phi }(x_{j}) & \;\;\text{scale\textbackslash{};and\textbackslash{};shift} \end{array}$$

**Figure 3.**
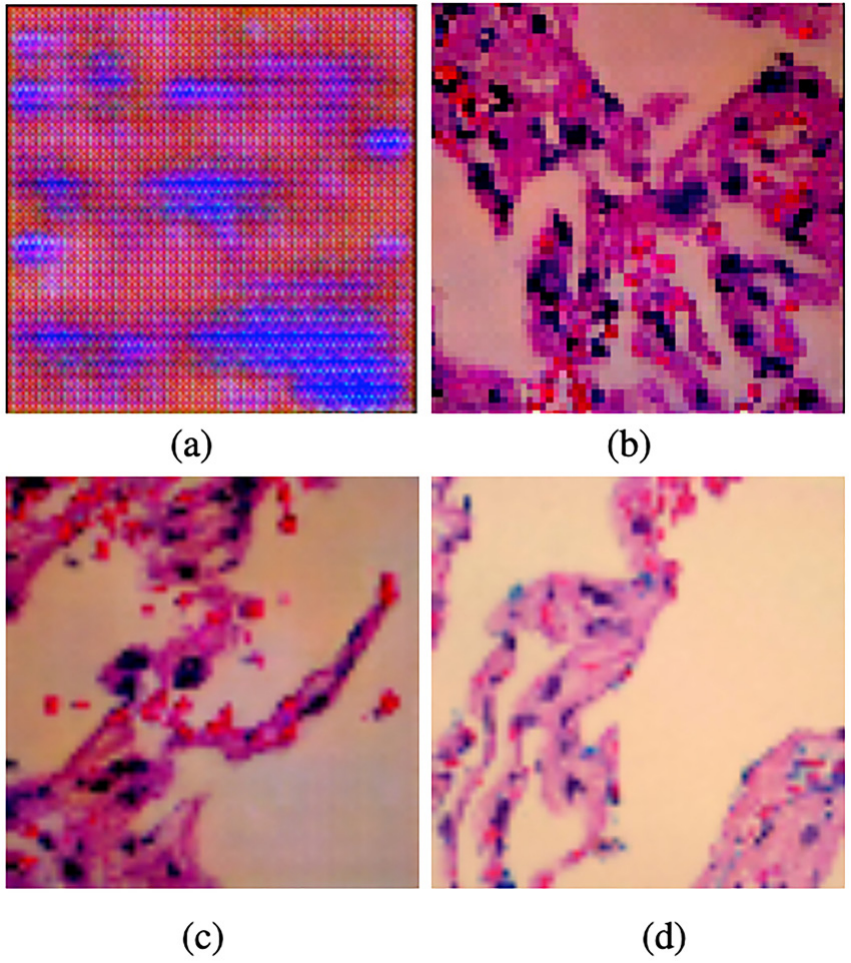
Comparison of lung benign images generated by the DCGAN, LDCGAN with original image. (a) DCGAN’s image, (b, c) artificial image, and (d) raw image.

Figure 3 compares different lung benign images. The image in Figure 3(a) is generated by the DCGAN, as the picture shows, the default setting of DCGAN will generate images with checkerboard patterns and blurry effects. When applying part settings of our DA-ENM, we find that the image in Figure 3(b) does not have checkerboard patterns. After applying all our settings, Figure 3(c) is more clear than Figure 3(b). By comparing the original image in Figure 3(d) and the image produced by the LDCGAN, the synthetic image no longer exists with checkerboard patterns and blurry effects.

After the additional settings beyond the original DCGAN, we resized the images of the raw dataset from 768×768 to 64×64 dimensions with a 256 batch size. When training the LDCGAN, we divided the dataset into 3 classes individually to train the LDCGAN, and we get three LDCGAN models that can synthesize different images for 3 lung cancer classes. Finally, we use LDCGAN to generate 10 000 artificial images for different lung cancer classes. These images are 64×64 pixels. Then we use these synthetic images combined with the resized original dataset (5000 images for each lung cancer class and overall 15 000 images) to produce a new dataset: 15 000 images for each lung cancer class, which contains 5000 original images and 10 000 synthetic images. In total, there are 45 000 images. The new dataset will be the dataset for training the DA-ENM (the second part of our LCDAE framework).

The first part of Figure 2 shows the overall architecture of our LDCGAN. Initially, we take random noise as the generator’s input and generate the synthetic images. Then we take these synthetic images and real images as input data to the discriminator. Finally, after finishing the training process of the LDCGAN, the generator can generate synthetic images close to the authentic images, and the discriminator cannot distinguish them from real images.

#### Data Augmented Ensemble Model DA-ENM

The DA-ENM includes 2 parts, the dataset preprocessing and the ensemble model. We implemented different data augmentation techniques to the dataset in the first part. In the second part, the ensemble model contains 3 parts: the pretrained CNNs, fine-tuning process, and the multimodel ensemble part. We will discuss the performance evaluation of DA-ENM in section “Results.”

##### Dataset Preprocessing

As we mentioned in section “Generative Model LDCGAN”, we produced a new dataset that contains 45 000 images in total for 3 different classes of lung cancer. Before we fed this dataset into the DA-ENM, we implement several data augmentation techniques to the current dataset.

**Figure 4.**
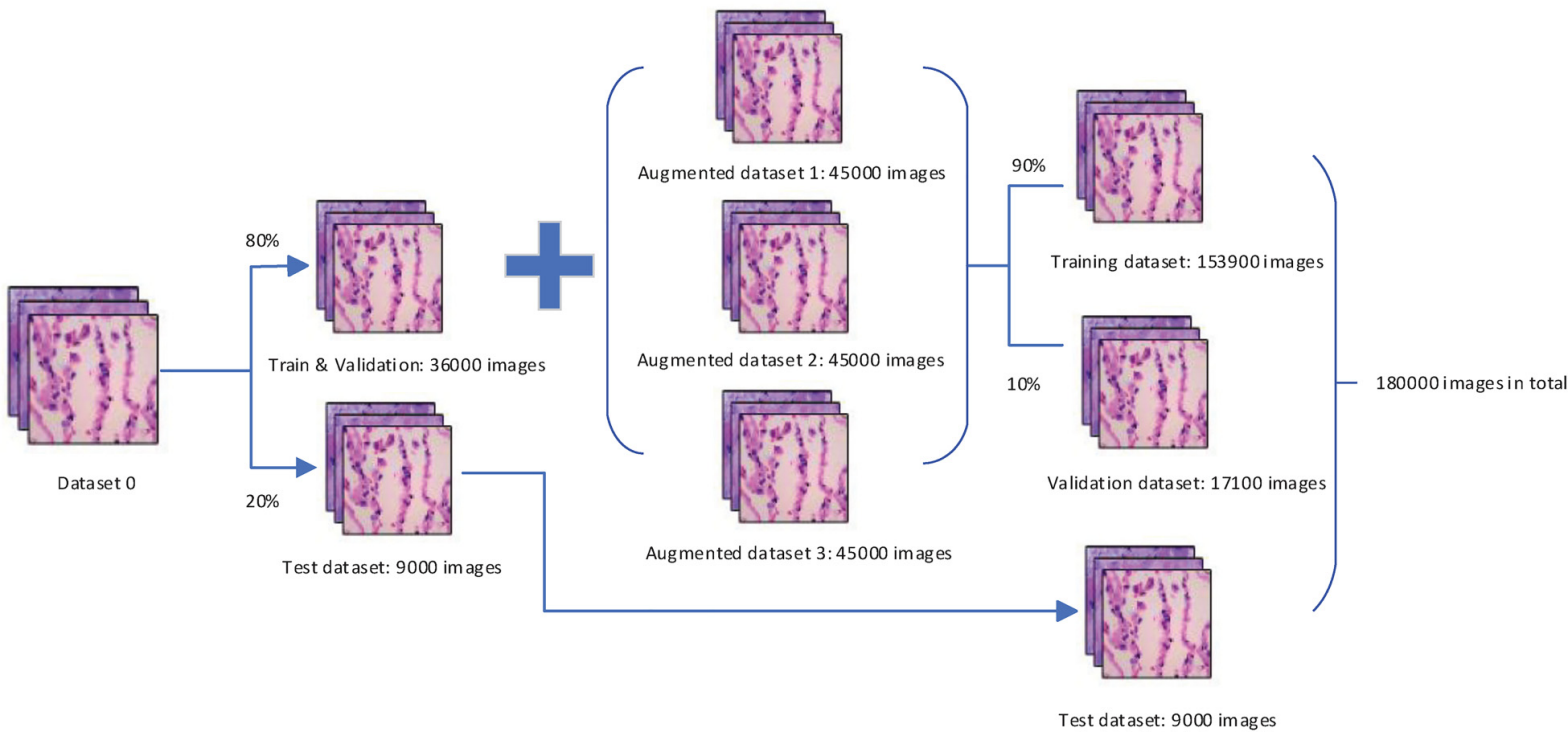
The final dataset after applying different data augmentation techniques.

As Figure 4 shows, we divide the dataset 0 (the dataset generated from the LDCGAN) into 2 sets, 20% for the test data and 80% for the train & validation data. Then we combine the train & validation dataset with 3 augmented datasets together to produce the new train & validation dataset. The data augmentation methods used in the 3 datasets are as follows:

- **Augmented Dataset 1**
  1. Change the value of the brightness, contrast, and saturation.
  2. Random rotation for all the images.
  3. Random affine transformation of the images and keep center invariant.
  4. Normalize data to shift the scale between 0 and 1 with mean and standard deviation.
- **Augmented Dataset 2**
  1. Random horizontal flip of the images.
  2. Random rotation of images.
  3. Random affine transformation of the images.
  4. Randomly erase a rectangle region for images.
  5. Normalization.
- **Augmented Dataset 3**
  1. Random horizontal flip ofthe images.
  2. Random rotation of images.
  3. Random affine transformation of data.
  4. Normalization.

After the dataset is produced by different data augmentation techniques mentioned above, we also do a global standardization. Initially, we calculate the mean of the dataset. Then we compute the standard deviation across all the channels within the entire dataset. The calculated mean and standard deviation values for this dataset are listed in Table 1.

**Table 1.** The Calculated Mean and Standard Deviation Across Each RGB Channel of the Entire Dataset.

Dataset	Channel	Mean	STD
Training dataset	Red	− 0.006	0.273
	Green	− 0.033	0.292
	Blue	0.105	0.468
Validation dataset	Red	− 0.006	0.274
	Green	− 0.033	0.293
	Blue	0.104	0.466
Test dataset	Red	0.013	0.249
	Green	− 0.019	0.279
	Blue	0.142	0.488

**Figure 5.**
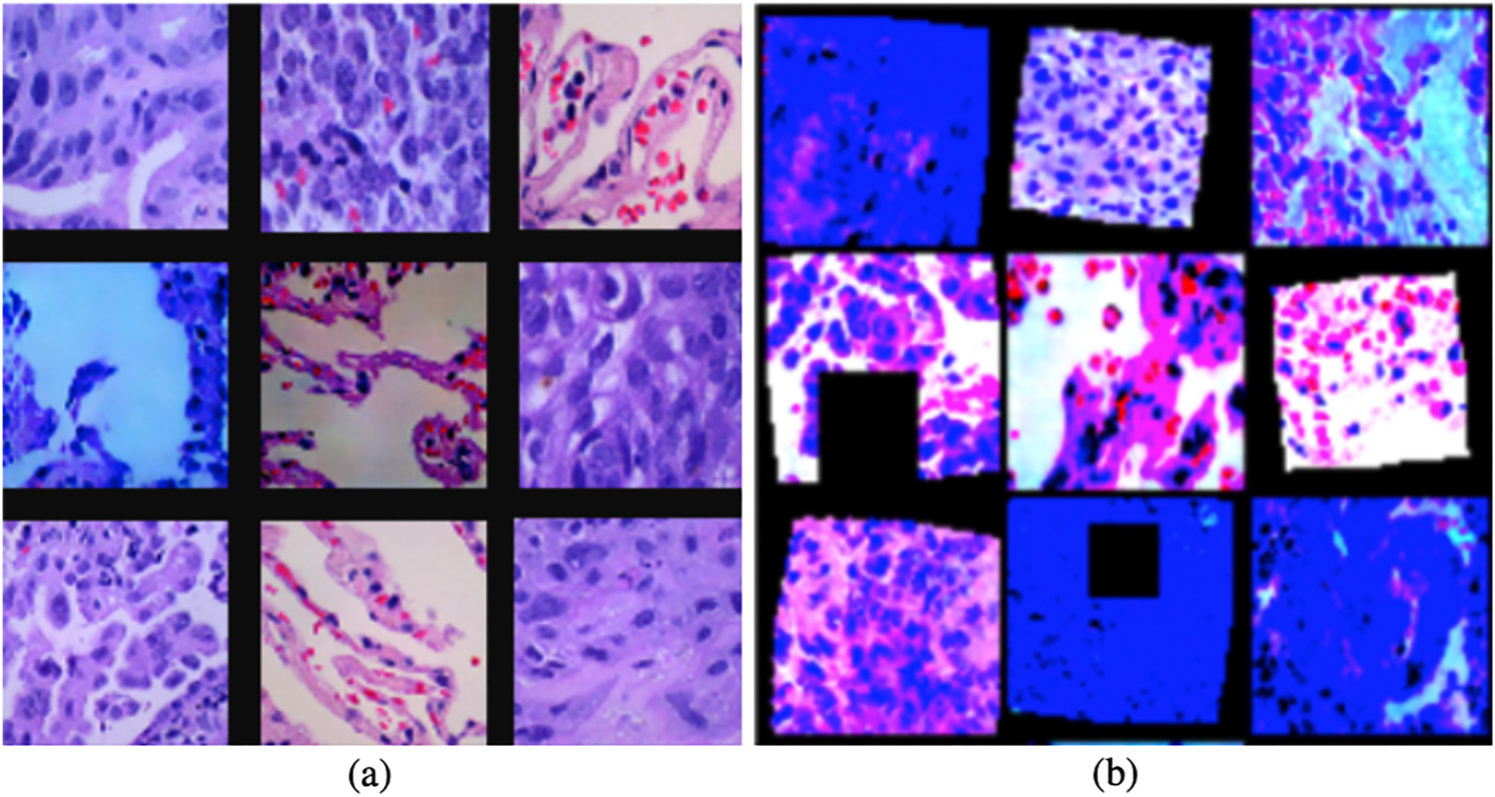
Comparison of original images and augmented images.

Figure 5 shows the differences between the original images and data augmented images. By comparing Figures 5(a) and 5(b), Figure 5(a) is a small set of the original dataset, and Figure 5(b) are images after applying different data augmentation techniques. We can easily find there are significant differences between them. Applying data augmentation techniques can help the model avoid overfitting problems. In the next step, we will use these data augmented datasets to train our DA-ENM model.

##### Ensemble Model

**Pretrained CNNs:** In the second part of the LCDAE framework, firstly, we use 6 different models with pretrained weights to do transfer learning. These models are DenseNet121,^45^ GoogLeNet,^42^ Resnet101,^43^ VGG19 with Batch Normalization,^44^ VGG16 with Batch Normalization,^44^ VGG16.^44^ All the pretrained models already show promising results for medical image classification tasks. When we load these pretrained models, we replace all the classifiers of these models. The last layer is replaced with a fully connected layer which has 3 output features. During the training process, we use the optimizer with adaptive moment estimation (Adam)^53^ with an initial learning rate of 0.001, then we will dynamically decrease the learning rate when the loss has stopped improving with the patience of 3 epochs and factor of 0.1. When we start the training process, we train the classifiers of each model, after finishing the training of classifiers, we do a fine-tuning process for each model to improve the performance. We will discuss this in the next paragraph.

**Fine-tuning:** Fine-tuning is an efficient technique that can outperform the feature extraction method. After we trained the classifiers for each model, we unfroze each model’s parameters to do a fine-tuning process. The fine-tuning can make the feature extraction phase of each model more suitable for the current dataset, and it can improve the performance of each model. During the training process of fine-tuning, we use the same optimizer and dynamic learning rate adjustment technique, which are the same as the previous process of training the classifiers. After the fine-tuning, we found that it improves the accuracy of the model and decreases the loss significantly. We will discuss the results in section “Results.” **Multimodel Ensemble:** When we finished the training process of pretrained model and fine-tuning. We concatenate the outputs for each model and add a new classifier to the final ensemble model. The classifier has a fully connected layer with a softmax function. The optimizer and the learning rate scheduler are the same as in the previous fine-tuning step. It needs to mention that we have not added more layers of the classifier in the ensemble model, according to the performances of pretrained and fine-tuned models, the overall performance is relatively high, then we just need to concatenate their outputs together and train a small classifier to get the final classification results.

The second part of Figure 2 shows the entire architecture of DA-ENM. Firstly, we load 6 original pretrained models to predict the results and we add classifiers to each model. Then we unfreeze all parameters of each model and do a fine-tuning process for each model. Finally, we combine the output of each model to get the final output.

#### Hybrid Data Augmentation

The hybrid data augmentation (HDA) contains all the data augmentation techniques used in the LCDAE. Firstly, the LDCGAN is 1 component of the HDA. As section “Data Augmentation” mentioned that the GAN is also a data augmentation technique. Moreover, the HDA also includes the methods shown in section “Dataset Preprocessing”, we summarize them as follows:

- Randomly change the value of the brightness, contrast, and saturation.
- Random rotation for all the images.
- Random affine transformation of the images and keep center invariant.
- Normalize data to shift the scale between 0 and 1 with mean and standard deviation.
- Random horizontal flip of the images.
- Randomly erase a rectangle region for images.

The HDA is an essential part of LCDAE. By combining these different data augmentation techniques with an ensemble model, then we can get the excellent performance of the LCDAE.

### Evaluation Methods

In this article, we measure the results of our LCDAE by a multiclassification confusion matrix. The confusion matrix contains accuracy, precision, sensitivity, and F1-score. For each class of lung cancer, we can calculate these indexes by equation (4). The TP refers to the value of true positive, the FP refers to the value of false positive, the FN refers to the value of false negative, and the TN indicates the value of true negative.

(4) $$\begin{array}{cc} {accuracy}_{j} & =\frac{{TP}_{j}+{TN}_{j}}{{TP}_{j}+{FP}_{j}+{FN}_{j}+{TN}_{j}}\\ {recall}_{j} & =\frac{{TP}_{j}}{{TP}_{j}+{FN}_{j}}\\ {precision}_{j} & =\frac{{TP}_{j}}{{TP}_{j}+{FP}_{j}}\\ F1\text{-}{score}_{j} & =\frac{2\times {precision}_{j}\times {recall}_{j}}{{precision}_{j}} \end{array}$$

To evaluate the overall performance of LCDAE, we use macroaveraged metrics to calculate them. The metrics include macroaveraged accuracy, precision, recall, and F1-score. Here, because the data contributions of each lung cancer class are the same, we use macroaveraged metrics instead of weighted-average metrics. The calculation of macroaveraged metrics is calculated by equation (5):

(5) $$\begin{array}{cc} {Accuracy}_{macro\_avg} & =\sum_{\;j=1}^{m}\frac{1}{m}\times {accuracy}_{j}\\ & =\sum_{\;j=1}^{m}\frac{1}{m}\times \frac{{TP}_{j}+{TN}_{j}}{{TP}_{j}+{FP}_{j}+{FN}_{j}+{TN}_{j}}\\ {Precision}_{macro\_avg} & =\sum_{\;j=1}^{m}\frac{1}{m}\times {precision}_{j}\\ & =\sum_{\;j=1}^{m}\frac{1}{m}\times \frac{{TP}_{j}}{{TP}_{j}+{FP}_{j}}\\ {Recall}_{macro\_avg} & =\sum_{\;j=1}^{m}\frac{1}{m}\times {recall}_{j}\\ & =\sum_{\;j=1}^{m}\frac{1}{m}\times \frac{{TP}_{j}}{{TP}_{j}+{FN}_{j}}\\ {F1\text{-}score}_{macro\_avg} & =\sum_{\;j=1}^{m}\frac{1}{m}\times {F1\text{-}score}_{j}\\ & =\sum_{\;j=1}^{m}\frac{1}{m}\times \frac{2\times {precision}_{j}\times {recall}_{j}}{{precision}_{j}} \end{array}$$

## Results

### Set-up of Experiments

The LDCGAN is training on an NVIDIA TESLA P100 GPU with 16 GB RAM. The CPU is Xeon with 13 GB RAM. The data augmentation of the original dataset was trained on the GTX 1070 8 GB GPU with 32 GB RAM. The data augmented ensemble model was trained on an A100 GPU with 80 GB RAM. In addition, the LDCGAN is running on the Keras^54^ framework, and other data augmentation techniques and DA-ENM were deployed by the PyTorch^55^ framework. Finally, we utilized the scikit-learn^56^ framework to generate our results.

### Results and Analysis

#### Images Synthesized by LDCGAN

**Figure 6.**
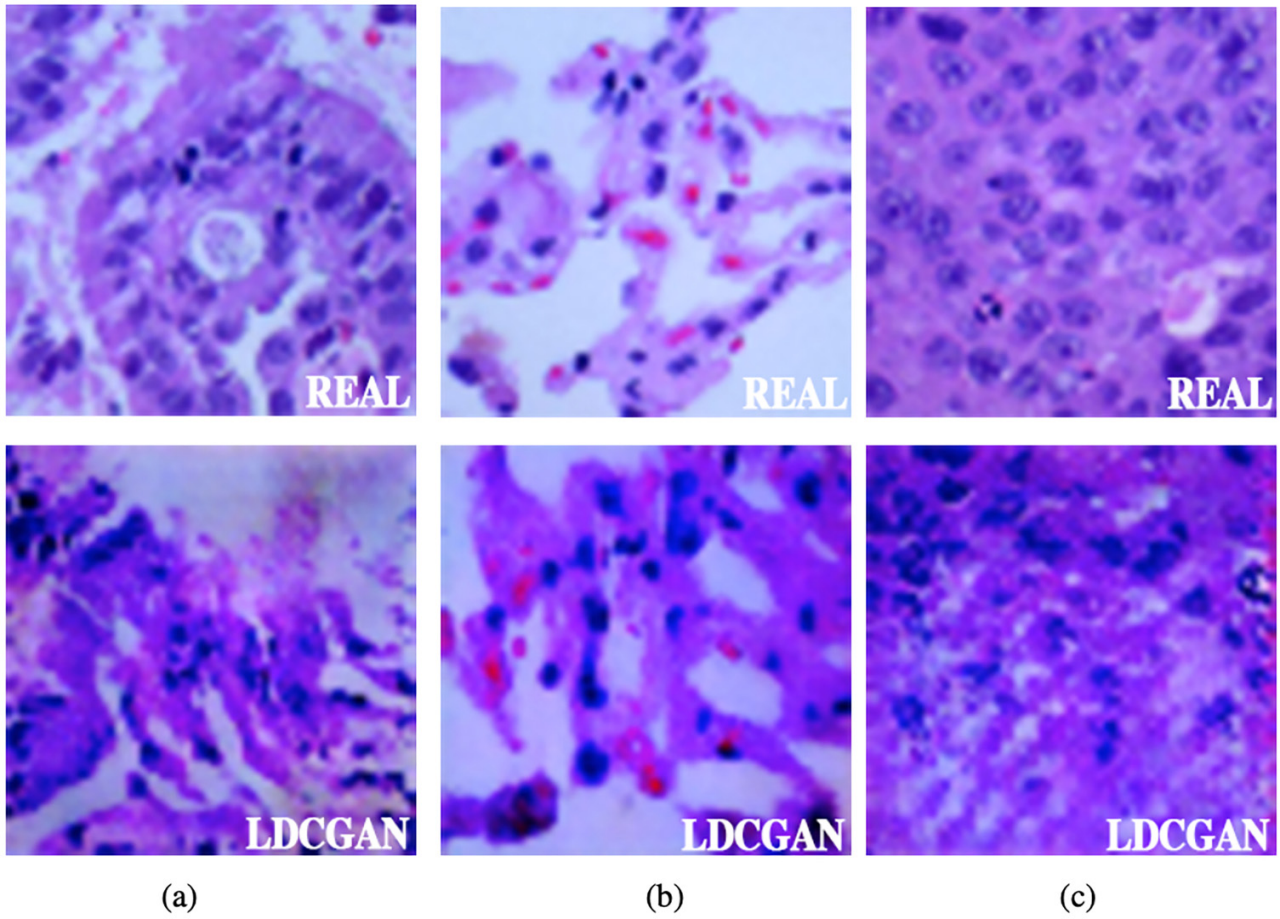
Compare LDCGAN Images with Raw Images.

The synthetic examples produced by the LDCGAN are shown in Figure 6. The images on the first row are from the original dataset and they are labeled as “REAL.” The images in the second row are synthetic images generated from LDCGAN, and they are labeled as “LDCGAN.” By comparison with “REAL” and “LDCGAN” images, we can find that the synthetic images inherit most of the features and patterns of the real images for all lung cancer classes. And they are difficult to distinguish from each other. After generating synthetic images, we feed the synthetic dataset and original dataset to our DA-ENM. Our results show that the synthetic dataset with other data augmentation techniques helped the DA-ENM achieve excellent results. We will discuss this in section “The Results of LCDAE”.

#### The Results of LCDAE

**Figure 7.**
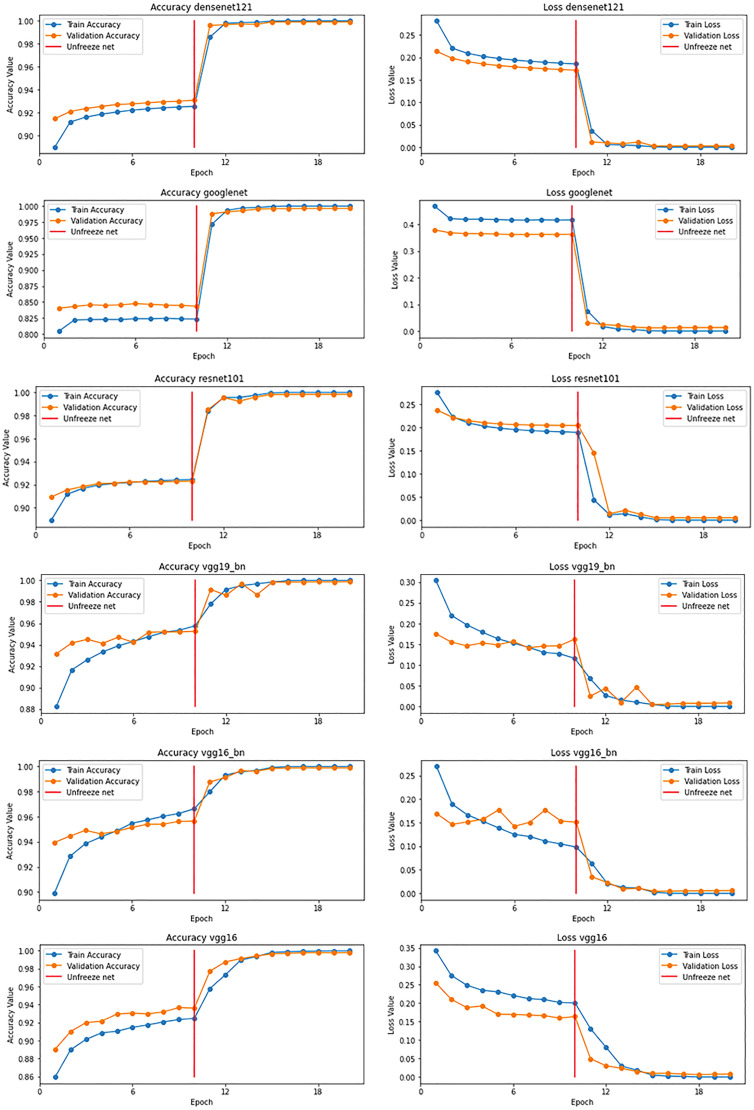
The performance of pretrained models after the fine-tuning process.

On our DA-ENM, we do a fine-tuning process for all the pretrained models before we implement the final multimodel ensemble. As Figure 7 shows, every model has 2 figures to describe the changes in accuracy and loss after the fine-tuning process. For each image, there is a red vertical bar, the left part of the red vertical bar illustrates the performance of the original pretrained model, and the right part of the red vertical bar shows the performance of the pretrained model after the fine-tuning process. We can see that there are apparent performance changes after the fine-tuning process. In Table 2, we show the accuracy improvements for each model: the accuracy of DenseNet121 increases by 7.21% after fine-tuning; the accuracy of GoogleNet rise by 14.96%, which is the highest increment after fine-tuning; the ResNet101 up to the 99.58%, which raises by the 7.6% of accuracy; and the VGG19-BN, VGG16-BN, and VGG16 grow by the 4.94%, 4,63%, and 6.97%, respectively. By analyzing the data in Figure 7 and Table 2, it is evident that the fine-tuning process increases the performance of all the models with pretrained weights, and the accuracies of all the models reached at least 99.35%. Moreover, the highest accuracy is 99.80% of DenseNet121. After the fine-tuning process for each model, we integrate all the models to increase the performance of the final ensemble model.

**Table 2.** The Improvements of Pretrained Models After Fine-tuning.

Model	Pretrained	Fine-tuning	Improvements
DenseNet121	92.59%	99.80%	7.21%
GoogleNet	84.48%	99.45%	14.96%
ResNet101	91.98%	99.58%	7.60%
VGG19_BN	94.57%	99.51%	4.94%
VGG16_BN	95.00%	99.64%	4.63%
VGG16	92.37%	99.35%	6.97%

Table 3 presents the results of LCDAE. The classification results of lung adenocarcinoma are listed as follows: 99.99% (accuracy), 100% (precision), 99.97% (recall), and 99.98% (F1-score); for lung benign, all the metrics are 100% except the accuracy of 99.99%; for lung squamous cell carcinoma, the accuracy of 99.99%, the precision is 99.97%, the recall is 100%, and the F1-score is 99.98%. In the last row of Table 3, we show the macroaveraged accuracy, precision, recall, and F1-score of LCDAE are 99.99%, respectively.

**Table 3.** The Results of LCDAE.

	Accuracy	Precision	Recall	F1-score
lung adenocarcinoma	99.99%	100%	99.97%	99.98%
lung benign	99.99%	100%	100%	100%
lung squamous cell carcinoma	99.99%	99.97%	100%	99.98%
macro avg	99.99%	99.99%	99.99%	99.99%

### Evaluate Performance with State-of-the-art Methods

In Table 4, we evaluate our approach with other state-of-the-art methods for the same dataset. Most of the methods have not used GAN as a data augmentation technique. They focus on the single CNN model to do classification. Our model combines the CNN model and multiple data augmentation techniques to get better performance than others.

**Table 4.** Compare the Performance of Different State-of-the-art Methods.

Author	Method	Accuracy	Precision	Sensitivity	F1-score
Bukhari et al.^58^	RESNET50	93.91%	95.74%	81.82%	96.26%
	RESNET18	93.04%	96.81%	84.21%	95.79%
	RESNET34	93.04%	95.74%	80.95%	95.74%
Phankokkruad^59^	Ensemble	91%	92%	91%	91%
	ResNet50V2	90%	91%	90%	90%
Hlavcheva et al.^60^	CNN-D	94.6%	-	-	-
Masud et al.^5^	DL-based CNN	96.33%	96.39%	96.37%	96.38%
Hatuwal and Thapa^61^	CNN	97.2%	97.33%	97.33%	97.33%
Mangal et al.^62^	Shallow-CNN	97.89%	-	-	-
Chehade et al.^63^	XGBoost	99.53%	99.33%	99.33%	99.33%
Abbas et al.^57^	Pre-ResNet101	99.8%	99.97%	100%	99.98%
Our method	**LCDAE**	**99.99%**	**99.99%**	**99.99%**	**99.99%**

The best-received result of other researchers is produced by Abbas et al.^57^ They used a pretrained ResNet101 model and got the results as follows: 99.8% (accuracy), 99.97% (precision), 100% (sensitivity), and 99.98% (F1-score). However, the accuracy, precision, and F1-score of our method are better than in Abbas et al.^57^ To the best of our knowledge, our method gets the best performance in this dataset.

### Generalization of Different Diseases

To evaluate the effectiveness of LCDAE, we also run LCDAE on the different public datasets. The results of LCDAE are shown in Table 5. Firstly, we evaluate the performance of the Brain Tumor MRI dataset,^64^ the dataset has 7022 images of human brain magnetic resonance imaging (MRI) images in total and images are classified into 4 classes: Glioma, Meningioma, No tumor, and Pituitary. LCDAE got the accuracy of 99.71%, the precision of 99.68%, the recall of 99.69%, and the F1-score of 99.69%. Secondly, the LCDAE is running on the Alzheimer dataset.^65^ This dataset contains 6330 Alzheimer MRI images with 3 classes: mild demented, nondemented, and very mild demented. The results of this dataset are the accuracy of 99.37%, the precision of 99.50%, the recall of 99.50%, and the F1-score of 99.50%. The last experiment tests on the COVID-19 dataset,^66^ which has 454 images with 3 classes: COVID-19, viral pneumonia, and normal. The results of the COVID-19 dataset are the accuracy of 98.44%, the precision of 98.15%, the recall of 97.92%, and the F1-score of 97.97%. By analyzing these results, LCDAE can also get excellent performance on other datasets.

**Table 5.** The Macroaverage Results of LCDAE on Different Datasets.

Datasets	Accuracy	Precision	Recall	F1-score
Brain Tumor MRI^64^	99.71%	99.68%	99.69%	99.69%
Alzheimer^65^	99.37%	99.50%	99.50%	99.50%
COVID-19^66^	98.44%	98.15%	97.92%	97.97%

## Discussion

In the present study, we proposed a Lung Cancer Data Augmented Ensemble LCDAE framework to classify lung cancer images. The framework contains a generative model LDCGAN, data augmented ensemble model DA-ENM , and a hybrid data augmentation (HDA). The LDCGAN can produce artificial lung cancer images to train the deep neural network as an additional training dataset. Moreover, these additional images also can help the model avoid overfitting problems and improve its performance. The DA-ENM uses multiple data augmentation techniques to improve the model’s generalizability. It also provides more training images to the model. The DA-ENM is an ensemble model, which combines 6 pretrained models: DenseNet121, GoogleNet, ResNe101, VGG19-BN, VGG16-BN, and VGG-16. The hybrid data augmentation HDA combines LDCGAN and all the data augmentation techniques used in the DA-ENM. Before building the ensemble model, we do a fine-tuning process for each model. Then DA-ENM combines the outputs from these models to make the final prediction. Finally, our framework obtains the results as follows: 99.99% (accuracy), 99.99% (precision), 99.99% (sensitivity), and 99.99% (F1-score). The obtained results show that our method can solve the overfitting problem, and it can get the best performance evaluated with other latest methods for lung cancer classification tasks.

In a study conducted by Bukhari et al^58^, they proposed 3 variants of CNN: ResNet18, ResNet34, and ResNet50. They got the highest accuracy (93.91%) of ResNet50. The ResNet30 and ResNet18 got an accuracy of 93.04% individually. Another study proposed by Phankokkruad^59^ uses an ensemble model which combines the outputs from VGG16, ResNet50V2, and DenseNet201. Their ensemble model achieved an accuracy of 91%. Chehade et al^63^ designed a machine learning model called XGBoost, this model is based on the feature engineering method known as unsharp masking. The results of XGBoost are as follows: 99.53% (accuracy), 99.33% (precision), 99.33% (recall), and 99.33% (F1-score). The best results for the current dataset are obtained by Abbas et al.^57^ They used a number of pretrained models to do lung cancer classification. These models are AlexNet, VGG19, ResNet18, ResNet-34, ResNet50, and ResNet101. The best results are 99.8% (accuracy), 99.97% (precision), 100% (recall), and 99.98% (F1-Score). By comparing these methods, our LCDAE is the only 1 using the GAN as a data augmentation technique with other possible data augmentation techniques. Our results show that the LDCGAN can be a powerful technique to produce synthetic images to improve the model’s performance. Moreover, our DA-ENM combines most of the efficient pretrained models to get the best performance. Furthermore, our method can efficiently overcome the overfitting issue when classifying lung cancer images. It gets the highest accuracy of 99.99%, the precision of 99.99%, and the F1-score of 99.99% compared with the latest approaches.

Although we achieved excellent performance and solved the overfitting of the lung cancer classification tasks, there are still some potential works that we need to improve in the future. These limitations are shown below.

1. There are still variances between synthetic images and original images.
2. We cannot generate different classes of lung cancer images by using 1 model, we have to train 3 separate models to generate 3 different classes of lung cancer images.
3. LDCGAN cannot generate high-resolution images. The images with high resolution are very important for the biomedical domain.
4. The ensemble model consumes much computation power and time to train.

We also plan the possible solutions for the limitations mentioned above. For the differences between synthetic and real images, we can try to use different loss functions to minimize the differences between them. For generating different classes of lung cancer images, we can try to use CGAN^67^ to solve the problem. The last limitation is generating high-resolution images. We can try to use StackGAN or its variants to synthesize high-resolution images. These potential solutions are working well in other domains. We will explore the performance of these potential solutions for lung cancer classification tasks.

## Conclusions

In this article, we developed a data augmented ensemble framework LCDAE, it includes a LDCGAN as a data augmentation technique, a data augmented ensemble model DA-ENM, and a hybrid data augmentation (HDA). Before we train the ensemble model, we use multiple data augmentation techniques in the HDA to increase the generalizability of the DA-ENMand avoid overfitting problems for the lung cancer classification tasks. By comparing with other latest methods, our approach reaches the best performance with 99.99% (accuracy), 99.99% (precision), 99.99% (sensitivity), and 99.99% (F1-score). The motivation comes from the fact that there are limited datasets in lung cancer classification tasks, and the deep learning models have potential risks of overfitting the source domain features with poor generalizability. Our framework can remedy this shortcoming and reach the highest performance.

## Abbreviations

LDCGAN: Lung Cancer Deep Convolutional GAN
LCDAE: Lung Cancer Data Augmented Ensemble
DA-ENM: Data Augmented Ensemble model
HDA: Hybrid Data Augmentation
CT: computed tomography
MRI: magnetic resonance imaging.
